# **Early parkinson’s disease: levodopa requirements are associated with the striatal DaT-uptake**

**DOI:** 10.1007/s00702-025-02999-9

**Published:** 2025-08-16

**Authors:** Katarina Rukavina, Nicola Mulholland, Ben Corcoran, Magdalena Krbot Skoric, Silvia Rota, Alexandra Rizos, K Ray Chaudhuri

**Affiliations:** 1 Movement Disorders Hospital Beelitz, Str. Nach Fichtenwalde 16, 14547 Beelitz-Heilstätten, Germany; 2 Parkinson Foundation Centre of Excellence, King’s College Hospital, London, UK; 3 Institute of Psychiatry, Psychology & Neuroscience at King’s College London, King’s College London, London, UK; 4 Department of Nuclear Medicine, King’s College Hospital NHS Foundation Trust, London, UK; 5 Department of Nuclear Medicine, Barts Health NHS Trust, London, UK; 6 Department of Neurology, University Hospital Center Zagreb, Zagreb, Croatia

**Keywords:** Clinical biomarkers, Personalized medicine, Levodopa requirements, Striatal dopaminergic deficiency

## Abstract

Precision medicine driven by clinical biomarkers is the state-of-art management approach for Parkinson's disease (PD). Whether pattern of striatal dopaminergic deficiency (demonstrated by single-photon emission CT (SPECT) scanning with ^123^I-Ioflupane, *DaTSCAN*) could be a biomarker predicting levodopa requirement in early PD is not known. Participants with early PD (disease duration (DD) ≤ 5 years, Hoehn and Yahr (H&Y) ≤ 3) who underwent DaTSCAN as a part of clinical-diagnostic work up and were enrolled in the “Non-motor Longitudinal International Study” (UK National Institute for Health Research Clinical Research Network Number 10084) were included in this cross-sectional analysis. Specific DaTSCAN binding ratios were analyzed for each striatum, caudate nucleus and putamen and the z-scores were derived normalizing the images to age and gender-matched healthy controls from the European-Database-of-DaTSCAN-of-healthy-controls (ENC-DAT). Using linear regression analysis, we explored the impact of DaT-uptake z-scores for more severely affected putamen, caudate nucleus and striatum on the LEDD. Statistically significant predictors identified in the univariable analysis were included in the multivariable analysis with DD and H&Y as additional independent variables. 43 PwP (30% female; age: 61.91 ± 11.45years; DD: 2(0–5) years; H&Y: 2(1–3); LEDD: 424.27 ± 342.62 mg) were assessed 19.12 ± 13.11 months following the DaTSCAN. In a multivariable linear regression analysis, when adjusted for DD and H&Y, z-caudate nucleus (B=-134.073, 95% CI -262.715 - -5.431, *p* = 0.042) and z-striatum (B=-162.137, 95% CI -306.306 - -17.967, *p* = 0.028), were statistically significant predictors of LEDD, while z-putamen was not (*p* = 0.086). In early PD, striatal DaT-uptake z-scores may serve as biomarkers that could aid the LEDD estimation and guide treatment decisions towards personalized care.

## Introduction

In Parkinson’s disease (PD), single-photon emission computed tomography (SPECT) brain imaging with a radiopharmaceutical DaTscan™ (123I-Ioflupane injection) is a helpful tool to assess the presence, extent and pattern of nigrostriatal dopaminergic depletion. (Bega et al. 2021) In addition to its supportive role in the differential diagnosis of non-dopamine deficiency aetiologies of movement disorders, DaTscan™ may potentially serve as a clinical biomarker for outcome prediction, since striatal DAT availability correlates with the burden of parkinsonian motor symptoms and striatal DAT binding decreases progressively with increasing disease severity. (Pirker 2003) (EMA) (Rukavina et al. 2023) In individuals with early stages of PD, dopamine deficiency in posterior putamen has been indicated as a key determinant of the severity of motor deficits (measured using the Unified PD Rating Scale Part III (UPDRS III)), while increasing severity of both anxiety symptoms and depression symptoms (measured using the State-Trait Anxiety Inventory (STAI) and Profile of Mood States Scale (POMS) depression subscale respectively) correlate with decreased DAT availability in the left anterior putamen region. (Jeong et al. 2022) (Liu et al. 2015) (Weintraub et al. 2005) Further, it has been shown that early caudate nucleus dopaminergic dysfunction might serve as a risk marker for rapid clinical disease progression and may predict the onset of cognitive impairment, depression and gait issues. (Pasquini et al. 2019)

Symptomatic treatment of PD relies on dopaminergic substitution, and precisely customizing its dose to tackle each individual’s unique needs (precision medicine) is key for successful symptom relief. However, in clinical practice, reliable and readily available neuroimaging and/or biochemical markers to aid the precise dose estimation are still lacking. (Titova and Chaudhuri 2017) Whether the extent and pattern of striatal dopaminergic deficiency demonstrated by DaTscan™could serve as an indicator aiding the estimation of levodopa equivalent daily dose (LEDD) requirements in early PD is not clear. Here, we hypothesized that in PwP at early stages of the disease, lower striatal dopaminergic availability visualized by DaTscan™ neuroimaging is associated with higher LEDD requirements.

## Materials and methods

We conducted an explorative, retrospective, cross-sectional analysis of data collected as a part of prospective, observational, multicenter, international study “*The Non-motor International Longitudinal*,* Real-Life Study in PD - NILS*” (authorized by the South East London Research Ethics Service (REC reference number 10/H0808/141) and adopted as a national study by the National Institute of Health Research in the United Kingdom (UK; UK National Institute for Health Research Clinical Research Network (UKCRN) No.10084). The NILS is a combined natural history and treatment effect-based study, where, using validated assessment tools, nonmotor symptoms (NMS) are evaluated on an annual basis to explore their evolvement over time, their response to the conventional treatment and their impact on the health-related quality of life. Individuals of all genders and all age groups, who have received diagnosis of PD based on the UK Brain Bank Criteria within the previous 5 years meet the NILS inclusion criteria, while those with atypical parkinsonism, concomitant severe disease, conditions interfering with PD assessments (as estimated by the treating neurologists) and those unable to give informed consent are not eligible, as described before. (Hughes et al. 1992) (van Wamelen et al. 2021) (van Wamelen et al. 2019) Written informed consent, including consent to use neuroimaging data for research purposes, was obtained from all participants prior to the inclusion in the study.

This analysis was conducted on the sample consisting of the NILS participants with early PD (disease duration ≤ 5 years, Hoehn and Yahr (H&Y) stage ≤ 3) recruited at the Movement Disorders Outpatient Clinics at the Parkinson’s Foundation Centre of Excellence at King’s College Hospital, London, United Kingdom in the period between 1st July 2020 and 31st December 2022 who underwent DaTSCAN as a part of their clinical-diagnostic work up.

### Assessments

Participants’ demographic (age and gender) and disease-related characteristics (disease duration, current medication) were collected through a structured interview and noted from the NILS database. LEDD was calculated for each participant. (Schade et al. 2020)

Clinical assessments analysed included:

*Hoehn and Yahr (H&Y) stage* – a 5-point scale for staging of PD-associated disability into 5 categories: unilateral disease (H&Y Stage I), bilateral disease with intact balance (H&Y Stage II), the presence of postural instability (H&Y Stage III), loss of physical independence (H&Y Stage IV) and being wheelchair- or bed-bound (H&Y Stage V). This practical staging method allows for reproducible assessments by independent examiners. (Hoehn and Yahr 1967)

*Short Parkinson’s Evaluation Scale (SPES)/SCales for Outcomes in Parkinson’s disease – Motor Function (SCOPA-Motor)* – a rater-based scale consisting of 21 items with four-response options (ranging from 0 (normal) to 3 (severe) organized in 3 sections: Motor Evaluation (Section A), Activities of Daily Living (Section B) and Motor Complications (Section C) (Martinez-Martin et al. 2005).

*The Parkinson’s Disease Questionnaire - Short Form (PDQ 8)* is a PD-specific measure of self-perceived health status consisting of 8 items rated with scores from 0 (“never”) to 4 (“always” or “cannot do at all”) and covering eight dimensions of ill-health. (Jenkinson and Fitzpatrick 2007)

Specific DaTSCAN striatal binding ratios (SBR) for striatum, the caudate nucleus and putamen were obtained from medical records. To account for age-expected decline in striatal binding, the z-scores were derived normalizing the images to age and gender-matched healthy controls from the European-Database-of-DaTSCAN-of-healthy-controls (ENC-DAT). (Varrone et al. 2013) The more affected side was defined as a side with lower z-scores. (Rukavina et al. 2023)

### Statistical analysis

Statistical analysis was run using the SPSS statistic software, version 26 (IBM). The normality of data distribution was tested (One-sample Kolmogorov–Smirnov test) and descriptive statistics provided. Using linear regression analysis, we explored the impact of z-scores for SBRs for more severely affected putamen, more severely affected caudate nucleus and more severely affected striatum on the LEDD. Statistically significant predictors identified in the univariate analysis were included in the multivariate analysis together with disease and H&Y stage as major clinical covariates. Statistical significance was set at *p* < 0.05.

## Results

43 individuals (30% women) with early PD were assessed over a mean 19.12 ± 13.11 months following the DaTSCAN. Their demographic and disease characteristic features, as well as their mean DaTSCAN z-scores for SBR in striatum, putamen and the caudate nucleus are presented in Table 1.

**Table 1:** Participants’ demographic and disease characteristic features

*N* = 43	
Age Mean ± SD; years	61.91 ± 11.45
Sex Female: n, %	13 (30%)
Hoehn&Yahr stage Median, Range	2 (1–3)
SCOPA-Motor scale a) total score b) Part I c) Part II d) Part III Mean ± SD / Median, Range	a) 11.88 ± 5.12 / 12 (3–25) b) 6.4 ± 2.78 / 6 (2–14) c) 4.51 ± 3.05 / 5 (0–12) d) 0.98 ± 1.37 / 0 (0–5)
Disease duration Median, Range; years	2 (0–5)
LEDD Mean ± SD, mg/24 h	424.27 ± 342.62
PDQ-8 total score Mean ± SD	6.95 ± 5.67
NMSS total score Mean ± SD / Median, Range	45.33 ± 32.59 / 39 (3–136)
Mean DaTSCAN z-scores for SBR Mean ± SD	Striatum: -2.99 ± 0.71 Putamen: -3.44 ± 0.6 The caudate nucleus: -2.45 ± 0.74

*SD –* standard deviation, *SCOPA- Motor* - Scales for Outcomes in Parkinson’s disease – Motor Function, *PDQ-8* – Parkinson’s Disease Questionnaire, short form, *NMSS* – Non-motor Symptoms Scale, *SBR* – striatal binding ratio

DaTSCAN z-scores for SBR differed significantly between the more and the less affected striatum, the more and the less affected putamen and the more and the less affected caudate nucleus. Mean z-scores for SBR in putamen were significantly lower than mean z-scores for SBR in the caudate nucleus. Table 2.

**Table 2:** Comparison of z-scores for striatal binding ratios between the more and the less affected striatum, the more and the less affected putamen and the more and the less affected caudate nucleus

	Difference (mean±SD)	95% CI	*p*
z-scores for SBR in more vs. less affected striatum	-0.73 ± 0.52	-0.894 – -0.574	< 0.001*
z-scores for SBR in more vs. less affected caudate nucleus	-0.66 ± 0.44	-0.790 – -0.521	< 0.001*
z-scores for SBR in more vs. less affected putamen	-0.32 ± 0.47	-0.461 – -0.173	< 0.001*
Mean z-scores for SBR in putamen vs. in the caudate nucleus	-0.98 ± 0.39	-1.101 – -0.861	< 0.001*

In addition, mean z-scores for SBR in putamen and in the caudate nucleus are compared

SD – standard deviation, SBR – striatal binding ratio

A p-value of less than 0.05 was considered statistically significant

In a set of univariate linear regression models, DaTSCAN z-scores for SBR in the more severely affected striatum (B = -189.430, 95% CI for B = -333.943 - -44.916, *p* = 0.011) and z-scores for SBR in the more severely affected caudate nucleus (B = -169.420, 95% CI for B = -298.964 - -39.876, *p* = 0.012) emerged as significant predictors of the LEDD. No significant association was noted between the DaT-uptake z-scores for putamen and the LEDD (*p* = 0.063).

**Fig. 1:**
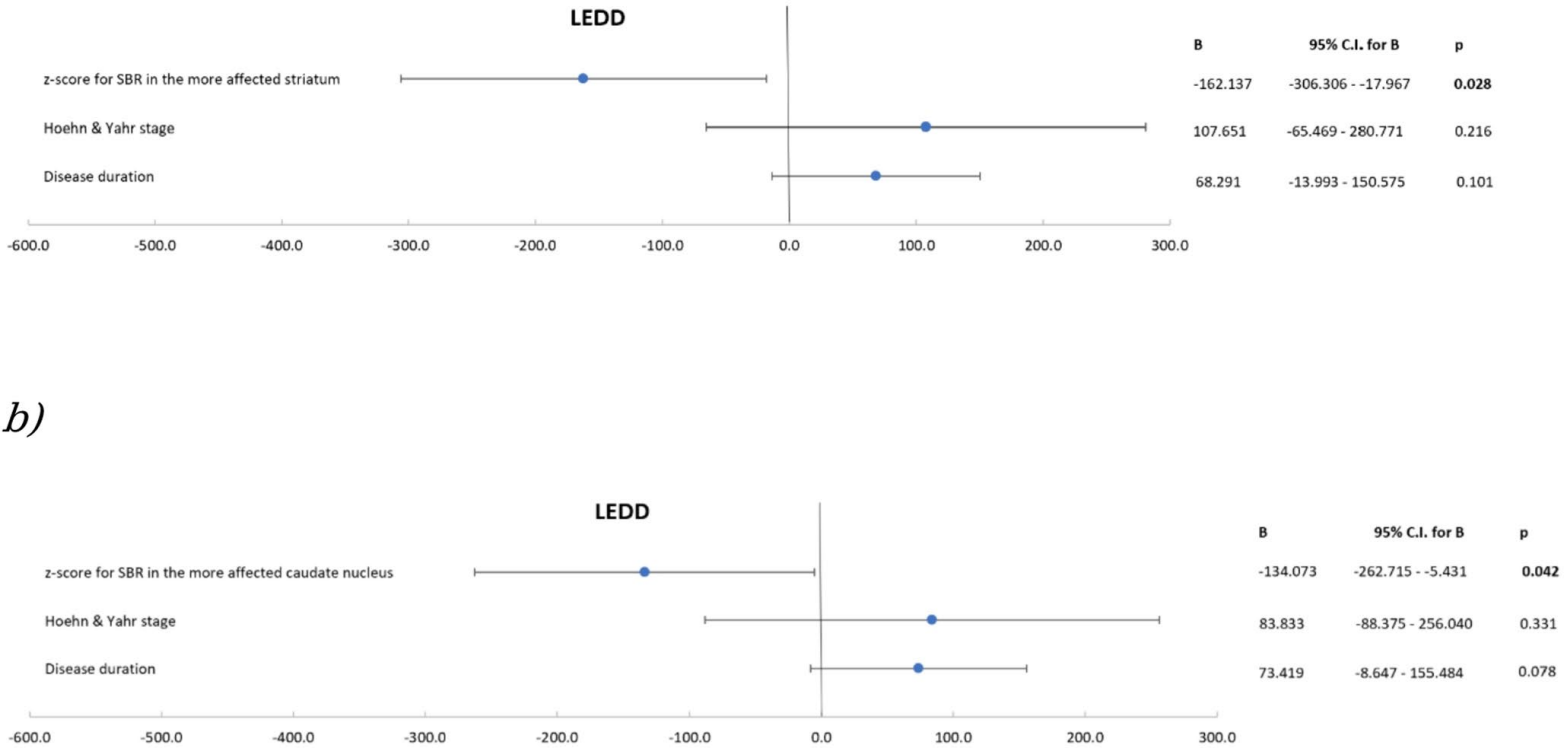
In a multivariate linear regression analysis, the DaTSCAN z-scores for SBR in (a) the more affected striatum and (b) the more affected caudate nucleus were, after the adjustment for disease duration and Hoehn and Yahr stage, significantly associated with the LEDD. *LEDD* – levodopa equivalent daily dose, *SBR* – striatal binding ratio

Following the adjustment for major clinical covariates (disease duration and H&Y stage) in a multivariate linear regression analysis, an association between the DaTSCAN z-scores for SBR in the more affected striatum (B=-162.137, 95% CI -306.306 - -17.967, *p* = 0.028. Figure 1. *A*) and LEDD, as well as between z-scores for SBR in the more affected caudate nucleus (B=-134.073, 95% CI -262.715 - -5.431, *p* = 0.042, Fig. 1. *B*) and LEDD retained statistical significance. Again, no association between z-scores for SBR in the more affected putamen and LEDD was noted (*p* = 0.086). Figure 1,

*A)*

## Discussion

Key finding of this small-sample, pilot analysis is a significant association between the extent of dopaminergic depletion in striatum, particularly in the caudate nucleus, visualized on routinely performed DaTSCAN™ images and LEDD requirements in early stages of PD. This relationship retained its statistical significance even after controlling for major clinical features, disease duration and H&Y stage. Despite the limitations of a retrospective data analysis, our findings suggest that striatal and the caudate nucleus BRs may possibly serve as approximate markers for LEDD requirement in early PD.

Neuropathologically, dopaminergic neuronal loss in the substantia nigra pars compacta has a central role underpinning the neurodegeneration that constitutes PD, while, in terms of symptomatic treatment, majority of approved pharmacological options aim to enhance dopaminergic neurotransmission. (Frasier et al. 2022) (Postuma et al. 2015; Titova et al. 2017) (Martinez-Martin et al. 2011) Correspondingly, our findings suggest that the degree of striatal dopaminergic depletion may impact the medication requirements. Of note, this is likely only relevant in PwP at early stages of the disease (such as in our cohort), whereas in later stages, involvement of multiple neurotransmitters becomes increasingly evident and emergence of late onset NMS and treatment-resistant motor symptoms makes management of PD more challenging. (Frasier et al. 2022) (Rukavina et al. 2021).

DaTSCAN serves as a diagnostic tool to visualize and quantify striatal dopaminergic decline in neuro-degenerative Parkinsonian syndromes, such as PD, and facilitate the differential diagnosis of non-dopamine deficiency aetiologias of Parkinsonism in cases of clinical uncertainties. (Bega et al. 2021) However, its role in prediction of motor and NMS burden, motor complications and disease severity remains complex and controversial. (Najmi et al. 2025)

Several neuroimaging studies revealed the associations between striatal DaT availability with severity of motor (Jeong et al. 2022) (Liu et al. 2015) (Pirker 2003) (Pasquini et al. 2019) and NMS of PD (Weintraub et al. 2005) (Liu et al. 2020) (Pasquini et al. 2019). There appear to be an inverse correlation between overall striatal DAT binding and global measures of disease severity such as the H&Y stage, the total score on the Unified Parkinson’s Disease Rating Scale (UPDRS), and UPDRS activities of daily living, with a progressive decline of DAT binding with increasing disability. (Najmi et al. 2025) In general, although mean striatal SBRs in PwP decrease over time, this decrease may not be optimal for use as a marker of disease progression on its own, as the effect of age-related or compensatory mechanisms or pharmacological manipulation cannot be excluded. (Au et al. 2005) Despite this, in a recent model-based analysis of the Parkinson’s Progression Marker Initiative (PPMI, 449 participants with early PD), distinctive subtypes were identified based on the inter-individual heterogeneity of SBRs decline rate. Within individual subtypes, longitudinal decline of DaTSCAN parameters for the putamen and the caudate nucleus was consistent with worsening of clinical motor scores as assessed by the MDS-UPDRS Part III. (Zhou et al. 2021) In another recent study using data from the PPMI database (*n* = 196), lower DaTSCAN SBR values were associated with more advanced disease severity, as measured by the H&Y stage. (Najmi et al. 2025)

Of note, in our analysis, DaTSCAN z-scores for SBR in the more affected caudate nucleus emerged as significant predictors of the LED. This is in line with observations from the PPMI cohort, where reduced DAT availability in the caudate nucleus shortly after diagnosis was associated with an elevated risk of clinical progression to cognitive impairment, depression and gait problems in the next four years. (Pasquini et al. 2019) In a recent analysis of the PPMI cohort (*n* = 196), PwP who exhibited dyskinesia, as assessed by the MDS-UPDRS Part III, showed significantly lower SBR values in the right caudate nucleus on DaTSCAN imaging.(Najmi et al. 2025).

In clinical practice, in addition to its diagnostic purposes, clinicians may consult DaTSCAN when making treatment decisions. For example, in a retrospective analysis of 455 individuals who received DaTSCAN under PD service of a single tertiary center, clinical management was changed following the DaTSCAN in 42% of participants. The changes included the initiation of dopaminergic medication (the most frequent change, in 63% of cases), or medication adjustements (31%). (Tsang and Walker 2023) In line with this, our findings suggest that the initial extent of the nigrostriatal dopaminergic pathways’ neurodegeneration might inform the selection of the optimal dose of dopaminergic medication for patients with early PD.

The main limitations of our study are its retrospective design and the sample size which is, due to the study’s exploratory, pilot nature and gathering of “real-life” neuroimaging data obtained in the course of the clinical diagnostic work up, rather limited. Due to the cross-sectional design of our study, longitudinal data are not available, including detailed clinical assessments at the time the DaTSCAN was performed. Similarly, information on whether participants were receiving dopaminergic medication at the time of the DaTSCAN is not available. However, previous research has demonstrated that dopaminergic treatment does not significantly influence DaTSCAN imaging results. (Schillaci et al. 2005) Therefore, it is unlikely that this limitation has affected the validity of our findings. Despite those limitations, we believe that the use of a widely available biomarker in a real-life population of PwP distinguishes our study, and that its findings are, while still requiring a confirmation in larger studies, highly relevant for clinical practice and may bring precision medicine nearer to PwP.

To conclude, the findings of our analysis of the data collected in a real-life clinical setting suggest that striatal (and particularly the caudate nucleus) DaT-uptake z-scores may serve as surrogate biomarkers that may aid the precise LEDD estimation in individuals with early PD. Nonetheless, LEDD estimation must always be grounded in thorough clinical evaluation and tailored to the specific needs of each individual living with PD. (Riederer et al. 2025)

## Data Availability

The data collected and analysed in this study are available from the corresponding author upon a reasonable request.
